# Genome-Wide Investigation of the Role of MicroRNAs in Desiccation Tolerance in the Resurrection Grass *Tripogon loliiformis*

**DOI:** 10.3390/plants7030068

**Published:** 2018-08-31

**Authors:** Isaac Njaci, Brett Williams, Claudia Castillo-González, Martin B. Dickman, Xiuren Zhang, Sagadevan Mundree

**Affiliations:** 1 Centre for Tropical Crops and Biocommodities, Queensland University of Technology, Brisbane, QLD 4000, Australia; injaci@gmail.com (I.N.); b.williams@qut.edu.au (B.W.); 2 Department of Biochemistry and Biophysics, Institute for Plant Genomics and Biotechnology, Texas A&M University, College Station, TX 77843, USA; castillo.cm@tamu.edu (C.C.-G.); xiuren.zhang@tamu.edu (X.Z.); 3 Department of Plant Pathology and Microbiology, Institute for Plant Genomics and Biotechnology, Texas A&M University, College Station, TX 77843, USA; mbdickman@tamu.edu

**Keywords:** microRNAs, dehydration, desiccation, resurrection plants, *Tripogon loliiformis*, post-transcriptional gene silencing, miRNAs

## Abstract

Drought causes approximately two-thirds of crop and yield loss worldwide. To sustain future generations, there is a need to develop robust crops with enhanced water use efficiency. Resurrection plants are naturally resilient and tolerate up to 95% water loss with the ability to revive upon watering. Stress is genetically encoded and resilient species may garner tolerance by tightly regulating the expression of stress-related genes. MicroRNAs (miRNAs) post-transcriptionally regulate development and other stress response processes in eukaryotes. However, their role in resurrection plant desiccation tolerance is poorly understood. In this study, small RNA sequencing and miRNA expression profiling was conducted using *Tripogon loliiformis* plants subjected to extreme water deficit conditions. Differentially expressed miRNA profiles, target mRNAs, and their regulatory processes were elucidated. Gene ontology enrichment analysis revealed that development, stress response, and regulation of programmed cell death biological processes; Oxidoreductase and hydrolyase molecular activities; and SPL, MYB, and WRKY transcription factors were targeted by miRNAs during dehydration stress, indicating the indispensable regulatory role of miRNAs in desiccation tolerance. This study provides insights into the molecular mechanisms of desiccation tolerance in the resurrection plant *T. loliiformis*. This information will be useful in devising strategies for crop improvement on enhanced drought tolerance and water use efficiency.

## 1. Introduction

The majority of higher plants are sensitive to dehydration and lose viability upon the loss of 41–70% of their total water content [1]. However, some plants are well adapted to adverse environments and implement adaptation mechanisms that mitigate the effects of water loss [2]. Although the adaptation mechanisms are effective under diverse environments, enabling plant survival, severe water loss still leads to the death of the plant. Resurrection plants represent a small but diverse group of angiosperms that exhibit unique tolerance mechanisms to cope with desiccation [3]. This unique group of plants can tolerate desiccation in their vegetative tissues for prolonged periods and rapidly recover their metabolic activity within 48 h of watering with minimal or non-existent tissue damage [3,4]. To achieve this phenomenal tolerance, resurrection plants utilize a repertoire of strategies that include the rapid shutdown of photosynthesis; leaf and cell wall structural adjustment; the stabilization of subcellular milieu through the accumulation of sugars, late embryogenesis abundant proteins (LEA), heat shock proteins, and other compatible solutes; and the induction of extensive antioxidant and ROS scavenging systems during desiccation [5,6,7,8].

*Tripogon loliiformis* is a small tufted diploid (2n = 20) annual to short-lived perennial C4 resurrection grass. It is endemic to a wide range of habitats in Australia and “resurrects” from its desiccated state within 72 h [9,10]. It’s short life cycle, ploidy level, and ease of propagation makes it a suitable experimental model for desiccation tolerance studies. *T. loliiformis* physiological responses to desiccation are characterized by structural, physiological, and biochemical changes that include leaf folding, cell wall folding and vacuole fragmentation, the early shutdown of photosynthesis, the retention of chlorophyll (homoiochlorophyllous) [9,11], increased anthocyanin accumulation, and the accumulation of sucrose and trehalose [12]. Response to stress is genetically encoded and transcriptional and post-transcriptional reprogramming are important elements in stress tolerance strategies [13,14,15,16]. Previous studies have linked miRNAs with many cellular processes [17,18].

MiRNAs are a class of 20–22 nt endogenous non-coding small RNAs that play important roles in the regulation of gene expression at the transcriptional and post-transcriptional level in animals, plants [19,20], and unicellular organisms [21]. In plants, miRNAs are involved in many metabolic and biological processes, where they play crucial regulatory roles in growth and development [22], phytohormone signaling [23], and adaptive responses to abiotic and biotic stress [17,24,25]. In Arabidopsis, using mannitol as a stress-inducing agent, miR396, miR168, miR167, and miR171 were found to be drought-responsive [26]. In rice, 30 differentially expressed miRNAs were identified under drought stress, while in tobacco, miR395 and miR169 were found to be sensitive to drought stress [27]. Using a combination of high throughput sequencing and microarray technology in a genome-wide study of *Populus euphratica*, Li et al. [28] identified 131 differentially expressed miRNAs under drought stress.

Although studies on miRNAs have been conducted in drought sensitive and tolerant plants including wheat [29], sorghum [30], switch grass [31], brachypodium [32], *Arabidopsis* [25], rice [33], and maize [34], their role in stress tolerance in desiccation tolerant plants has not been investigated. While drought sensitive and tolerant plants employ tolerance mechanisms to withstand mild to moderate dehydration stress, it is postulated that resurrection plants utilize a repertoire of unique desiccation tolerance mechanisms to overcome and adapt to extreme conditions. In this study, *T. loliiformis* plants subjected to desiccation stress at strategic dehydration, desiccation, and rehydration stages were analyzed through high throughput sequencing for the identification and enrichment of conserved miRNAs and elucidation of their potential roles in desiccation tolerance. Future crop production is threatened by the effects of global climate change, erratic weather patterns, and population growth. There is an urgent need for the development of climate resilient and water use efficient crops. Understanding the unique desiccation tolerance mechanisms harbored by resurrection plants presents a great potential for the development of robust crops.

## 2. Results

### 2.1. Analysis of Small RNAs in Shoot and Root of Tripogon loliiformis

**Figure 1:**
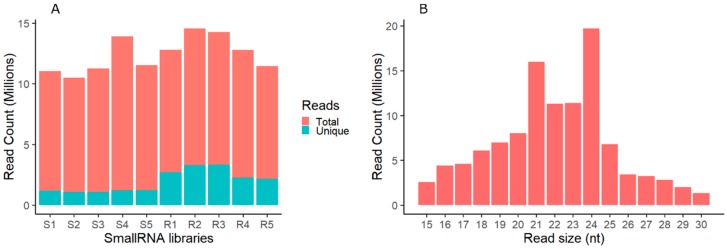
*Tripogon loliiformis* small RNA reads distribution. (**A**) Pre-processed reads distribution in shoot and root small RNA libraries; (**B**) overall reads distribution by size. S1–S5, R1–R5 represent shoot and root libraries at Hydrated (1), 60% RWC (2), 40% RWC (3), <10% RWC (4), and 48 h after rehydration (5), respectively.

The unique desiccation tolerance capabilities of resurrection plants suggests underlying tolerance mechanisms and effective regulation of transcription and translation. Studies have shown that miRNAs regulate gene expression at the transcriptional and post-transcriptional level. To investigate the role of miRNAs in desiccation tolerance, small RNA libraries from shoots and roots of hydrated, dehydrating (60% and 40% Relative Water Content (RWC)), desiccated (<10% RWC), and rehydrated plants were sequenced and the identification and expression profiling of miRNAs were conducted. High-throughput sequencing generated 142 million small RNAs reads in the range of 13–16 million reads per library, with an average of 1–3 million unique reads (Figure 1A). The raw reads were pre-processed to remove adapter sequences, low quality reads, and <15 nt reads, resulting in 104 (73%) million clean reads that were used for downstream analysis. Consistent with previous studies, the majority of the small RNAs were in the range of 21–24 nt, with 24 nt having the most reads, followed by 21 nt, across all libraries (Figure 1B).

### 2.2. Evolutionary Conservation of MiRNAs in Tripogon loliiformis

Many miRNAs are evolutionary conserved in species within the same kingdom and miRNA genes in one species may exist as orthologs or homologs in other species, presenting a powerful strategy to identify new miRNAs through a homology search [35]. To identify conserved miRNAs in *T. loliiformis*, the small RNA sequences were mapped against known plant miRNAs in the miRBase 21.0 database [36] and a total of 265 unique conserved miRNAs comprising 668 family members and isoforms from 60 MIR families were identified (Supplemental Table S1). Many of the identified miRNAs were family members and isoforms of the nine most evolutionary conserved miRNAs in plants [37]. The majority of the identified miRNAs were from closely related monocot species including *Oryza sativa*, *Sorghum bicolor*, *Zea mays*, *Branchypodium distachyon*, *Hordeum vulgare*, *Triticum aestivum*, and *Aegilops tauschii*, as well as from the model species *Arabidopsis thaliana* (Supplemental Figure S1).

### 2.3. Spatiotemporal Expression of MiRNAs in Tripogon loliiformis Tissues

**Figure 2:**
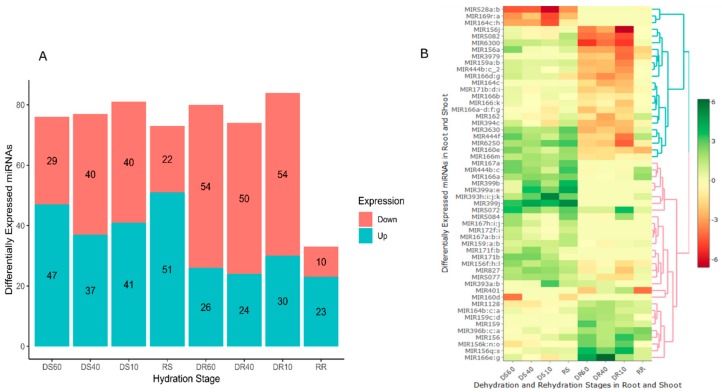
Differentially expressed miRNAs in shoots and roots under dehydration stress. (**A**) Bar graph showing disparate expression between shoot and roots. (**B**) Heatmap showing differentially expressed genes between shoots and roots at the different dehydration, desiccation, and rehydration stages. DS: Dehydrated Shoot, DR: Dehydrated Root, RS: Rehydrated Shoot, RR: Rehydrated Root.

Previous studies suggest that resurrection plants are genetically primed to respond to dehydration, even at a hydrated state, through the constitutive expression of stress response mechanisms [38,39]. Do resurrection plants gain their tolerance by post-transcriptionally regulating transcription more tightly than sensitive plants? Differential expression analysis identified 183 conserved miRNAs that differentially accumulated in shoots and roots during dehydration (Supplemental Table S2). A higher number of miRNAs were up-regulated in the shoots compared to the roots (Figure 2A). In the shoots at 60% RWC, 47 miRNAs were up-regulated and 29 down-regulated. At 40% RWC, 37 miRNAs were up-regulated while 40 were down-regulated. Surprisingly, miRNA accumulation was observed in desiccated tissue at <10% RWC, where 41 miRNAs showed an increased abundance, while 40 displayed a decreased accumulation. In rehydrating shoots, 51 miRNAs were up-regulated while 22 were down-regulated. In contrast, a reduced accumulation of miRNAs was observed in the roots where a higher number showed downregulation as dehydration ensued. In summary, 26 miRNAs were up-regulated and 54 down-regulated at 60% RWC, 24 up-regulated and 50 down-regulated at 40% RWC, and 30 up- and 54 down-regulated at <10% RWC, while 23 and 10 miRNAs were up- and down-regulated respectively on rehydration (Figure 2A, Supplemental Figure S2).

Tissue specific expression was observed where some miRNAs exhibited disparate expression between shoots and roots (Figure 2B). For example, miR399b, miR399j, miR167a, and miR393 h:i:j:k were up-regulated, while miR164c:h, miR169r:a, miR528a:b, and miR160d were down-regulated in the shoots, but were not expressed in the roots. The miRNAs miR444f, miR160e, and miR6300 were up-regulated in the shoots and down-regulated in the roots (Figure 2A), while other miRNAs showed a similar expression trend.

### 2.4. Tripogon loliiformis Stress-Associated MiRNAs Targets

MicroRNAs exert their post-transcriptional gene silencing role through base complementarity pairing with their cognate mRNA transcripts, leading to cleavage or translation repression. A large number of gene targets were predicted from *T. loliiformis* contigs annotated from RNAseq data generated in the same experiment [40], *S. italica* coding sequences, and *A. thaliana* transcripts preloaded in the psRNATarget genomic library for target prediction. A total of 1236 unique contigs targeted by conserved miRNAs were predicted in *T. loliiformis* (Supplemental Table S3). The majority of the predicted targets were orthologs of known conserved miRNAs targets involved in a broad range of biological processes including metabolism, response to abiotic stress, post-transcriptional gene silencing, regulation of development, and gene expression. The targets included transcription factors (TFs), protein kinases, transporters, chaperones, antioxidants, and carbohydrate metabolism associated genes. The transcription factors and other genes targeted by the miRNAs were interpreted to be differentially expressed based on the accumulation of their cognate miRNAs. The TFs associated with down-regulated miRNAs included members of Auxin response factor (ARF 5, 13, 22) targeted by miR162a, miR160a, and miR164b; *Squamosa* promoter binding-like protein (SPL 17, 18, 19) targeted by miR157d and miR156c; MYB (miR396, miR1128, miR159a), MADs-box (miR157a, 164b, miR1128), NAC (miR390), bHLH (miR408), and WRKY 38, 39, 70 targeted by miR167a, miR395b, and miR390. The TFs associated with up-regulated miRNAs included GATA 12 (miR396a), Scarecrow (miR171c), WRKY 4, 14 (miR5139, miR2916), ethylene-responsive transcription factor (ERF1) (miR5021), AP2/EREBP (miR166), bZIP (miR5021), and the nuclear factor Y (NFY A, C) as targets of miR169b and miR167a, respectively. However, it is worth noting that some members of the same transcription factor family, for example, WRKY, SPL, and MYB, showed disparate expression (Table S3). The predicted chaperones and heat shock proteins and factors targeted by miRNAs included HSP70, HSP90, and DNAJ. The antioxidants ascorbate peroxidase 4-like, peroxidase, and glutathione-s-transferase were among the targets for down-regulated miRNAs. A number of carbohydrate metabolism associated gene targets with a low accumulation of their cognate miRNAs included sucrose synthase (miR195a), Galactinol-sucrose galactosyltransferase (miR1128), fructokinase-4-like (miR319), hexokinase (miR319g:l, miR319a-d:f:h), and sucrose-phosphate synthase (miR167c) (Table S3). The accumulation of sucrose during dehydration and decline in glucose and fructose levels has been previously reported in a number of desiccation tolerant plants, including *T. loliiformis* [12,41].

The majority of the miRNAs targeting protein kinases were down-regulated during dehydration, indicating the active involvement of kinases in stress response signaling. The predicted kinases included class members of calcium-dependent protein kinase 1, 2, 3 targeted by miR157, miR169p, and miR159a, and CBL-interacting protein kinase as a target of miR408e. The expression of some kinases such as cysteine-rich receptor-like protein kinase (miR156h), MAP kinase (miR166h), serine/threonine-protein kinase (miR529), and phosphatidylinositol 4-kinase (miR408) was enhanced, while family members of some kinases displayed disparate expression. Transport associated members of ABC transporters, aquaporin NIP and H^+^ antiporter, were targeted by down-regulated miRNAs, while the K^+^ antiporter and aquaporin TIP and SIP were targets of up-regulated miRNAs during dehydration stress (Table S3).

### 2.5. Functional Roles of MiRNA Targets

MiRNAs control various cellular physiological, biochemical, and molecular processes. To investigate the potential functions and biological relevance of the predicted miRNA target genes, Gene ontology (GO) and enrichment analysis was conducted using Blast2GO [42] and GO terms viewed on Cytoscape [43]. The over-represented GO terms for the up-regulated miRNA gene targets included development associated processes such as cell development, growth and regulation of cell morphogenesis, metabolic and DNA catabolic processes, and regulation of programmed cell death. Other processes were related to vesicle fusion, membrane fusion, and protein transport and localization (Supplemental Figure S3). Over-represented metabolic activities included oxidoreductase, hydrolyase, nuclease, and ligase. The biological processes associated with down-regulated miRNA target genes included stress responses, defense and innate immunity, lipid and fatty acids metabolism, membrane transport, gene expression regulation, and post-transcriptional gene silencing (Supplemental Figure S4).

## 3. Discussion

MiRNAs research has been extensively conducted and their critical role in many biological processes enumerated [17,24,25,44]. In plants, most miRNA studies have focused on drought sensitive and tolerant species and no studies on desiccation responsive miRNAs in desiccation tolerant plants have been reported. Drought sensitive and tolerant plants employ stress tolerance mechanisms geared towards the restoration of homeostasis through water retention, albeit at different efficacies. Although resurrection plants utilize similar mechanisms at mild and moderate dehydration stress, they are primed for desiccation and utilize additional desiccation tolerance mechanisms to limit cellular damage to repairable levels through maintenance of the cellular structure and physiological integrity [2]. Based on the different response strategies, it is plausible that resurrection plants employ distinct regulatory processes at the post-transcriptional level that may not be observed in other vascular plant species. In this study, we performed a genome-wide profiling of miRNAs and their expression and targets in the desiccation tolerant *T. loliiformis*. High-throughput sequencing of small RNA libraries confirmed the evolutionary conservation of miRNAs through the identification of conserved miRNAs in *T. loliiformis*.

The high number of miRNAs identified suggests the critical role of miRNAs in gene expression regulation and their indispensable position in desiccation tolerance strategies in resurrection plants. The elevated accumulation of miRNAs in desiccated tissues suggested the existence of enhanced regulatory activities as dehydration ensued. However, the presence of miRNAs at a desiccated state could imply the repackaging and storage of miRNAs as the tissues dehydrated below 40% RWC, evidenced by high numbers during rehydration (Figure 2). The high number of miRNAs expressed during rehydration could be attributed to the regulation of cellular processes for protection against rehydration associated damage observed in resurrection plants [45].

Recent transcriptomics studies in resurrection plants indicated transcripts expression at extreme dehydration and attributed signal transduction proteins and retroelements to the instrumental role of gene silencing during desiccation [13,14,46]. In *T. loliiformis*, a similar trend was observed, where miRNAs expression was recorded at a desiccated state. Tissue specific expression was observed where miRNAs were induced in shoots and their expression was suppressed in roots (Figure 2). The expression disparity pointed to an inherent stress adaptation strategy where miRNAs act as master modulators of processes associated with energy metabolism, growth, and development in the shoots, while redirecting resources to promote stress tolerance and enhance protective mechanisms, some of which could be targeted to the roots. In Arabidopsis miR393, miR390/159, and miR159, TIR1, TCP/MYB, and SBP-LIKE, respectively, were induced under biotic and abiotic stress to suppress development-related processes for morphological adaptation to stress [17]. In resurrection plants, transcriptional reprogramming redirecting resources from growth processes towards cellular protection has been previously reported [13].

The majority of plant miRNAs target transcription factors that bind to conserved *cis*-acting promoter elements to affect the gene expression response, particularly those induced by abiotic stress [47]. Among the predicted transcription factors associated with down-regulated miRNAs during desiccation in *T. loliiformis* were nuclear factor Y NF-YA, NF-YB, and NF-YC targeted by miR169m, miR528, and miR167a, respectively. NF-Y is a CCAAT-DNA binding transcription factor that imparts significant tolerance to drought and facilitates increased yields in corn [48]. Members of the squamosa promoter-binding-like proteins (SPL) involved in the temporal regulation of shoot development and phase transition were differentially expressed, as previously reported [49]. The SPL transcription factors SPL17 (miR156a/c) and SPL18 (miR157) were cognates for down-regulated miRNAs and SPL6 (miR166), SPL12 (miR157), and SPL15 (miR5059) were predicted to be down-regulated. Under water deficit conditions, SPL are usually down-regulated as growth and developmental-associated processes including shoot development, vegetative phase transition, flowering, and leaf polarity are normally shutdown [17]. The observed differential expression implies that some members of SPL have stress tolerance-related functions in *T. loliiformis*, as recently reported in wheat [50]. The predicted members of the WRKY transcription factors modulate many processes in plants, including the response to abiotic and biotic stress [51]. The expression of WRKY 4, 38, 39, and 70 showed enhanced expression under extreme dehydration, pointing to their abiotic stress regulatory roles. In Arabidopsis, WRKY 70 was implicated in the positive regulation of defence, negative regulation of senescence, and modulation of osmotic stress tolerance through the regulation of stomatal aperture [52,53]. In the resurrection plant *Haberlea rhodopensis*, WRKY transcription factors were induced during desiccation [13]. The observed delayed senescence and early photosynthesis shutdown in *T. loliiformis* could be attributed to the regulation of WRKY 70 by miR390. Recent studies have associated NAC TFs with an unfolded protein response (UPR) in the ER stress response [54], while the regulation of GAMYB and ARF by miR159 and miR160 has been reported [49]. 

Protein synthesis and folding machinery in the endoplasmic reticulum (ER) can be compromised under unfavorable conditions, resulting in ER stress that activates the UPR [55,56]. The ER resident molecular chaperones play a critical role in the folding of newly synthesized proteins, maintenance of proteome integrity, and protein homeostasis [57]. In *T. loliiformis*, DNAJ and calnexin chaperones were predicted to be targeted by miR408 and miR5021, respectively. Calnexin (Cnx), an integral membrane protein, coordinates the processing of newly synthesized N-linked glycoproteins. DNAJ, on the other hand, binds directly on the luminal binding protein BiP, an HSP70 molecular chaperone that interacts with the newly synthesized polypeptides [58]. The observed down-regulation of miR408 and miR5021 would enhance the accumulation of DNAJ and calnexin chaperones to counter the ER stress during dehydration stress in *T. loliiformis*. Heterologous expression of *Oryza sativa* calnexin in tobacco conferred dehydration tolerance under mannitol stress [59].

Carbohydrate metabolism is a key process observed in resurrection plants during desiccation [7,60]. The accumulation of sucrose, oligosaccharides, and compatible solutes such as Late Embryogenesis Abundant (LEA) proteins and small heat shock proteins during desiccation leads to cellular stabilization through cytosolic vitrification [61]. Desiccation-induced accumulation of non-reducing trehalose sugar in *T. loliiformis* was recently associated with the induction of the cytoprotective autophagy pathways [12]. Comparative metabolic analysis of sucrose accumulation between desiccation tolerant and sensitive *Eragrostis nindensis* confirmed the crucial role of sucrose in desiccation tolerance, as sucrose only accumulated in the leaves of tolerant species [62]. Sucrose accumulation correlated with the expression of carbohydrate metabolic enzymes during desiccation, as previously reported in the resurrection plants *Craterostigma plantagineum* and *Haberlea rhodopensis* [13]. In *T. loliiformis*, the observed down-regulation of miRNAs targeting sucrose synthesis genes could lead to the enhanced expression of transcripts encoding sucrose synthase, sucrose 6-phosphate synthase, sucrose transporter, and galactinol synthase.

The lack of cellular damage and severe oxidative stress suggests elaborate protective mechanisms involving energy metabolism, growth, and development programs that regulate the induction of stress response mechanisms to provide a cushion against water loss. Differential miRNA expression analysis indicated distinct tissue specific accumulation patterns between the shoots and roots under desiccation stress. The majority of miRNAs were up-regulated in the shoots and down-regulated in the roots. For example, miR528a/b predicted to target LEA proteins and Calmodulin-like proteins (CML21) was down-regulated in shoots and had no expression in the roots. The accumulation of LEA proteins as a stress response mechanism has been previously reported. LEA proteins were reported to accumulate in shoot and scutellar, but not in root tissue of desiccation tolerant wheat seedling [63]. A variety of stress responses are mediated by Ca^2+^ signaling. For example, in rice, the multi-stress responsive gene2 (OsMSR2) was induced by multiple abiotic stress stimuli, and its overexpression in Arabidopsis enhanced tolerance to drought and salinity [64]. Chlorophyll biosynthesis is controlled by the scarecrow SCL27 gene through the regulation of photochlorophyllide oxidoreductase [65]. In *T. loliiformis*, miR171b predicted to target SCL27 was up-regulated in shoots and had zero expression in the roots. Disparate miRNA expression has been previously reported in other plants under diverse stress conditions [17], but not under desiccation tolerance. The observed spatiotemporal miRNAs expression in *T. loliiformis* suggests a unique adaptation strategy where extensive regulatory processes take place in the shoots, including early photosynthesis shutdown, induction of antioxidant systems, stress signaling, and resources mobilized and redirected to the roots to enhance survival.

## 4. Materials and Methods

### 4.1. Plant Materials and Stress Treatments

*Tripogon loliiformis* seeds from a single plant originally collected from Charleville in South western Queensland, Australia, were germinated in 65 mm plastic pots containing 50% native red soil and seed sowing potting mix in a growth chamber at 27 °C and for a 16 h photoperiod. The *Tripogon loliiformis* biological cycle completes in 12–14 weeks after germination. The plants are in their vegetative stage between two to six weeks and reproductive stage from the seventh to eighth week [66]. For this study, the dehydration experiment was conducted at the sixth week when the plants were in the vegetative stage. Twenty four hours prior to dehydration, fifteen pots containing multiple plants were well-watered to saturation. Three replicate samples from the hydrated plants were randomly collected. Since *T. loliiformis* is a small plant of approximately 5 cm in height, the entire aerial part (shoots) and roots, except the corm, were sampled per plant. The remaining plants were dehydrated by withholding water until they were air dry and their RWC dropped below 10% after six days [9]. During dehydration, triplicate shoot and root samples were collected at 60%, 40%, and <10% RWC. The collected samples were snap frozen in liquid nitrogen and stored at −80 °C until RNA extraction. Desiccated plants were watered and rehydrated samples collected after 48 h. The percentage RWC was determined using the leaf tissues and calculated according to Barrs, Weatherley [67]. *T. loliiformis* leaves were weighed upon sampling to get the fresh weight (FW). The leaves were placed in petri dishes containing water in a 4 °C fridge for 4–6 h. The leaves were blotted dry with a paper towel and weighed to get their turgid weight (TW). The leaves were dried overnight in a vacuum oven at 80 °C. The following day, the samples were cooled at room temperature in an aspirator to avoid error due to condensation, after which the dry weight (DW) was weighed. The percentage RWC of the leaf tissue was calculated using the formula

RWC (%) = ((Fresh Weight − Dry Weight)/(Turgid Weight − Dry Weight)) × 100.

### 4.2. Total RNA Extraction and High Throughput Sequencing

Total RNA was extracted from shoot and root tissues harvested from hydrated, dehydrating (60%, 40% RWC), desiccated (<10% RWC), and rehydrated plants using the Trizol Reagent (Invitrogen) according to the manufacturer’s instructions with modifications. Briefly, 50 mg of sample was ground in liquid nitrogen and transferred into a microfuge tube containing 1 mL of Trizol reagent, mixed thoroughly, and incubated for 5 min at room temperature. A total of 200 µL of chloroform was added, mixed by vortex, and centrifuged for 15 min at 4 °C, 14,000 rpm. Then, 350 µL of the top aqueous phase was homogenized in a Qiagen QIAshredder spin column and centrifuged at maximum speed for 2 min, and the flow-through was transferred into a new 2 mL collection tube for ethanol precipitation by adding 0.5 times 96% ethanol. SmallRNAs enriched total RNA bidding was done by transferring the mixture into a miRNeasy™ minElute™ spin column. Total RNA quality and purity were checked with the NanoDrop^®^ 2000 spectrophotometer (Thermo Scientific, Wilmington, DE, USA) and the integrity and quality were verified using a Bioanalyser (Agilent technologies, Santa Clara, CA, USA) (Supplemental Figure S5). Five triplicate shoot and root small RNAs libraries were prepared using the TruSeq™ Small RNA Sample Preparation protocol from Illumina^®^ and 50 bp single-end reads sequenced at Texas A&M AgriLife Genomics and Bioinformatics service, USA, using an Illumina HiSeq 2500 Sequencer (Illumina Inc., San Diego, CA, USA). The reads have been deposited in the Sequence Read Archive (SRA) at NCBI, Accession number SRP113187 (https://www.ncbi.nlm.nih.gov/Traces/study/?acc=SRP113187).

### 4.3. Small RNAs Data Analysis and MiRNAs Identification

Small RNA reads were pre-processed for quality and trimmed for removal of the 3′ adapter sequences using CLC genomics workbench 6.52 [68]. The pre-processed reads were mapped against the plant miRNA sequences in the miRBase release 21 [36] using CLC genomics workbench 6.52 [68]. Due to the absence of *T. loliiformis* genomic information at the time of this study, annotated miRNA sequences from 71 plant species in miRBase [36] were used as proxy references against which the miRNAs were annotated. A mismatch allowance of two was used in the conserved miRNA search. The 5′ mature sequences in the size range of 20–22 nt were filtered from the resultant hits as potential conserved miRNAs.

### 4.4. MiRNAs Expression Analysis

The expression profiles of identified miRNAs were determined using CLC genomics Workbench. The raw small RNA reads were mapped against the identified miRNAs to determine miRNA expression values in the shoot and root tissues across respective dehydration and rehydration states. To determine the differential expression of miRNAs throughout dehydration, desiccation, and rehydration, the expression values were enumerated and normalized against total read counts reported as reads per million (RPM). Using the hydrated stage as the control, all hydration stages were compared to identify the miRNAs that were constitutively expressed, induced, or suppressed. MiRNAs with a fold change of ±2 and a Bonferroni and FDR corrected *p*-value ≤ 0.05 were considered significant.

### 4.5. MiRNAs Targets and Their Functional Enrichment

Gene targets of the conserved miRNAs were predicted using the plant small RNA Target Analysis Server, psRNATarget [69], with the following parameters; maximum expectation (0–5):3, length of complementarity (15–30):19, and maximum energy to un-pair the target site (UPE, 0–100):25. The *Tripogon loliiformis* de novo assembled transcriptome dataset [40] and mRNA sequences from the closely related *S. italica*, as well as Arabidopsis, were used for the targets prediction. To identify the functional categories of the differentially expressed miRNA targets (Table 1), *T. loliiformis* transcriptome functional mapping encompassing all the plant metabolic pathways and enzyme functions was developed using the MapMan Mercator tool and visualization of predicted miRNA targets against the mapping was conducted using MapMan [70]. For Gene Ontology (GO) enrichment analysis, a BLAST search of *T. loliiformis* contigs against NCBI non-redundant (nr) Plantae sequences was conducted. The BLAST output was analyzed for GO enrichment with the Fischer exact test and a *p*-value of 0.05 to identify over-represented GO terms using Blast2GO [42]. The results were visualized using the Cytoscape BiNGO plugin [43].

## 5. Conclusions

Molecular studies have highlighted significant differences between resurrection plants and other dehydration sensitive species at the gene expression level. Some of the observed differences could be attributed to the post-transcriptional regulatory role of miRNAs. In this study, the results obtained suggest that *T. loliiformis*, a desiccation tolerant grass, elicits significant stress responses at the post-transcriptional level. *T. loliiformis* survival under extreme conditions could be attributed to efficient regulatory processes. Our findings in this study will enhance the understanding of *T. loliiformis* miRNA-mediated regulatory mechanisms and provide a foundation for further investigation of the gene pool of resurrection plants that could be holding many answers to agricultural crops.

## Figures and Tables

**Table 1:** MapMan functional categories of potential targets of differentially expressed conserved miRNAs during dehydration in *Tripogon loliiformis*.

Name	Contig Number	Accession Number	Target Description
**ABIOTIC STRESS RESPONSE**			
MIR408	Contig_1263	XP_004951273.1	BAG family molecular chaperone regulator 6
MIR172j	Contig_4654	EMS50802.1	DnaJ homolog subfamily C member 7
MIR3630	Contig_195	CAA47948.2	heat shock protein 70
MIR6250	Contig_29066	XP_003578630.1	hydrophobic protein OSR8-like
MIR166d	Contig_10495	XP_003557818.1	probable methyltransferase PMT2-like
**CARBOHYDRATE METABOLISM**			
MIR319a	Contig_91996	XP_003568782.1	hexokinase-7-like
MIR172h	Contig_914	XP_004984086.1	sucrose synthase 2-like
MIR167c	Contig_12310	XP_004965756.1	sucrose-phosphate synthase 3-like
MIR394c	Contig_1	XP_003566746.1	callose synthase 7-like
MIR166b	Contig_1239	XP_004984579.1	galactinol synthase 2-like
MIR167e	Contig_3855	XP_004958079.1	xylose isomerase-like
**DEVELOPMENT ASSOCIATED**			
MIR5021	Contig_16313	XP_004966491.1	senescence-associated protein DIN1-like
MIR172b	Contig_2082	XP_004962343.1	serine/threonine-protein kinase TOR-like
MIR528	Contig_16524	XP_004951509.1	serine-threonine kinase receptor protein
MIR156c	Contig_10275	XP_004957197.1	squamosa promoter-binding-like protein 17-like
MIR156	Contig_10275	XP_004957197.1	squamosa promoter-binding-like protein 17-like
**HORMONE METABOLISM**			
MIR160d	Contig_39233	EMT29346.1	1-aminocyclopropane-1-carboxylate synthase 7
MIR167h	Contig_16664	XP_004961973.1	ABSCISIC ACID-INSENSITIVE 5-like protein
MIR164a	Contig_14909	XP_004985140.1	Allene oxide synthase 2-like
MIR172b	Contig_975	XP_004956401.1	auxin transport protein BIG-like
MIR171e	Contig_10826	XP_004976258.1	IAA-amino acid hydrolase ILR1-like 5-like
**DNA REPAIR/SYNTHESIS/CHROMATIN STRUCTURE**			
MIR166h	Contig_3556	XP_003576514.1	DNA mismatch repair protein Msh6-1-like
MIR167a	Contig_548	XP_004975516.1	DNA repair helicase XPB1-like
MIR172a	Contig_49068	NP_001151792.1	DNA binding protein
MIR319g:l	Contig_5793	NP_001065884.1	HUA enhancer 2
MIR399a:e	Contig_961	XP_004961835.1	probable histone H2A.4-like

## Supplementary Materials

The following are available online at http://www.mdpi.com/2223-7747/7/3/68/s1, Figure S1: Annotation of *Tripogon loliiformis* conserved miRNAs using miRBase database, Figure S2: Cross-comparison Venn diagram showing the number of differentially expressed genes between shoots and roots, Figure S3: Gene Ontology of overrepresented GO terms associated with the targets of down-regulated miRNAs, Figure S4: Gene Ontology of overrepresented GO terms associated with the targets of up-regulated miRNAs, Figure S5: Representative Electrophoresis assay image of total RNA used for high-throughput sequencing. RNA with RNA Integrity Number (RIN) above 7.5 was used for sequencing library preparation, Table S1: List of conserved miRNAs identified in *Tripogon loliiformis*, Table S2: List of differential expressed conserved miRNAs in shoots and roots during dehydration, Table S3: List of predicted miRNAs targets associated with *T. loliiformis* contigs.
