# Supplementary material for: Genome-Wide Investigation of the Role of MicroRNAs in Desiccation Tolerance in the Resurrection Grass Tripogon loliiformis
Source: Plants (Basel). 2018 Aug 31;7(3):68. doi: 10.3390/plants7030068 (PMC6161015; doi:10.3390/plants7030068)
Supplement: Supplementary file 1 [file plants-07-00068-s001.zip › supplementary_figures/supplementary_figures_caption.docx]

**Figure S1:** Annotation of *Tripogon loliiformis* conserved miRNAs using miRBase database. MiRNAs abundance in *T. loliiformis* closely related species used to annotate the miRNAs*.* **Osa**: *Oryza sativa*, **Sbi**: *Sorghum bicolor*, **Zma**: *Zea mays*, **Bdi**: *Branchypodium distachyon*, **Hvu**: *Hordeum vulgare*, **Tae**: *Triticum aestivum*, **Ata**: *Aegilops tauschii*, **Ath**: *Arabidopsis thaliana*

**Figure S2:** Cross-comparison Venn diagram showing the number of differentially expressed genes between shoots and roots at the different dehydration, desiccation and rehydration stages. **a**) Comparison of up-regulated miRNAs between shoot and roots **b**) Comparison of down-regulated miRNAs between shoot and roots. **DS**: dehydrated shoot, **DR**: dehydrated root, **RR**: Rehydration Root, **RS**: Rehydration Shoot

**Figure S3**: Gene Ontology analysis showing overrepresented GO terms associated with the targets of down-regulated miRNAs in *T. loliiformis.*

**Figure S4**: Gene Ontology analysis showing overrepresented GO terms associated with the targets of up-regulated miRNAs in *T. loliiformis.*

**Figure S5**: Representative Electrophoresis assay image of total RNA used for high-throughput sequencing*.* RNA with RNA Integrity Number (RIN) above 7.5 was used for sequencing library preparation.

**Table S1:** List of conserved miRNAs identified in *Tripogon loliiformis****.*** Small RNAs analysis identified 265 known miRNAs comprising 668 isoforms and family members of 60 MIR families.

**Table S2**: List of differentially expressed miRNAs in *T. loliiformis* during dehydration

**Table S3**: List of predicted miRNAs targets associated with *T. loliiformis* contigs
