# Supplementary figures and images for: Genome-Wide Investigation of the Role of MicroRNAs in Desiccation Tolerance in the Resurrection Grass Tripogon loliiformis
Source: Plants (Basel). 2018 Aug 31;7(3):68. doi: 10.3390/plants7030068 (PMC6161015; doi:10.3390/plants7030068)

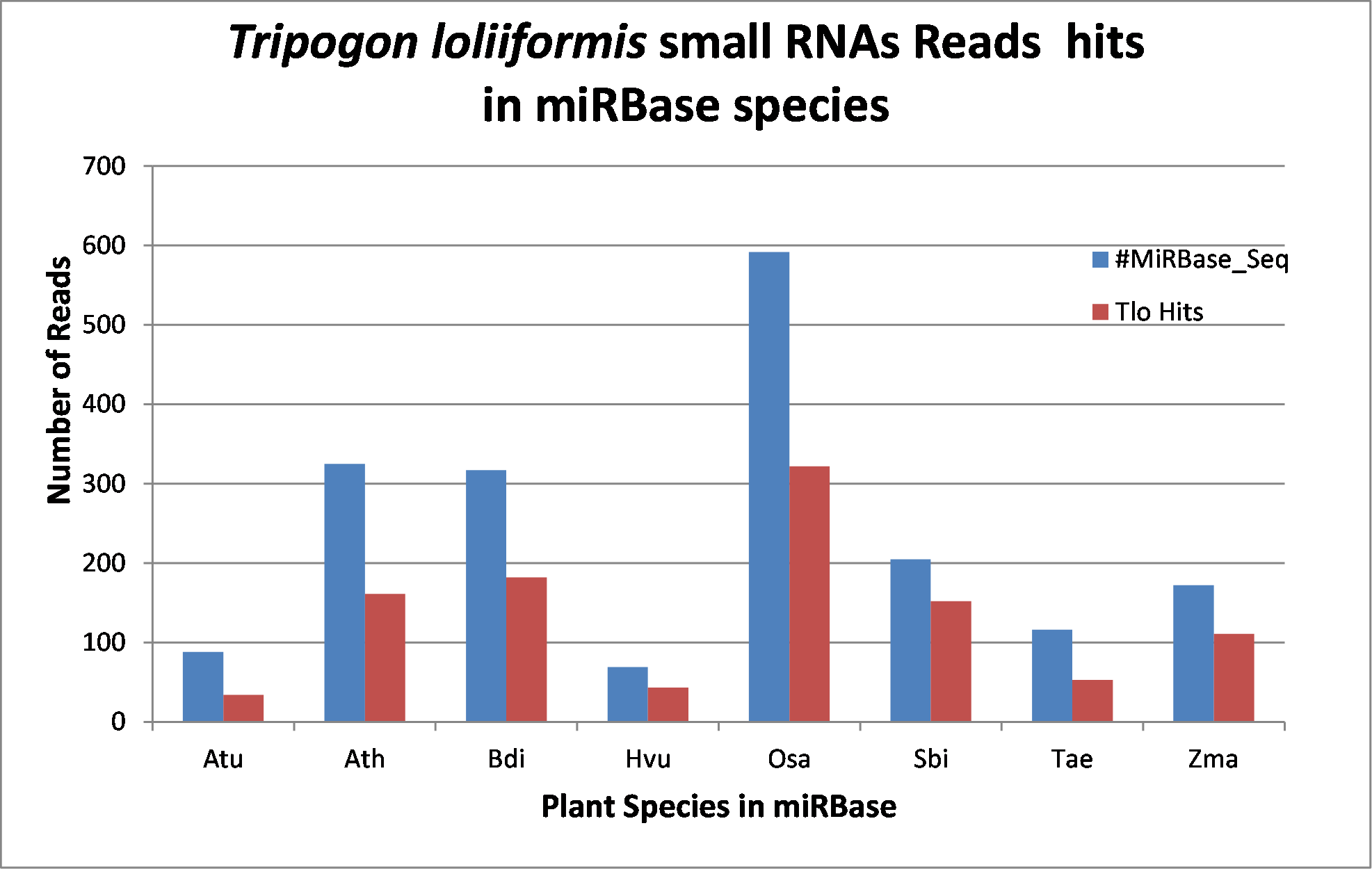

Supplement: Supplementary file 1 [file plants-07-00068-s001.zip › supplementary_figures/Figure S1.tif]

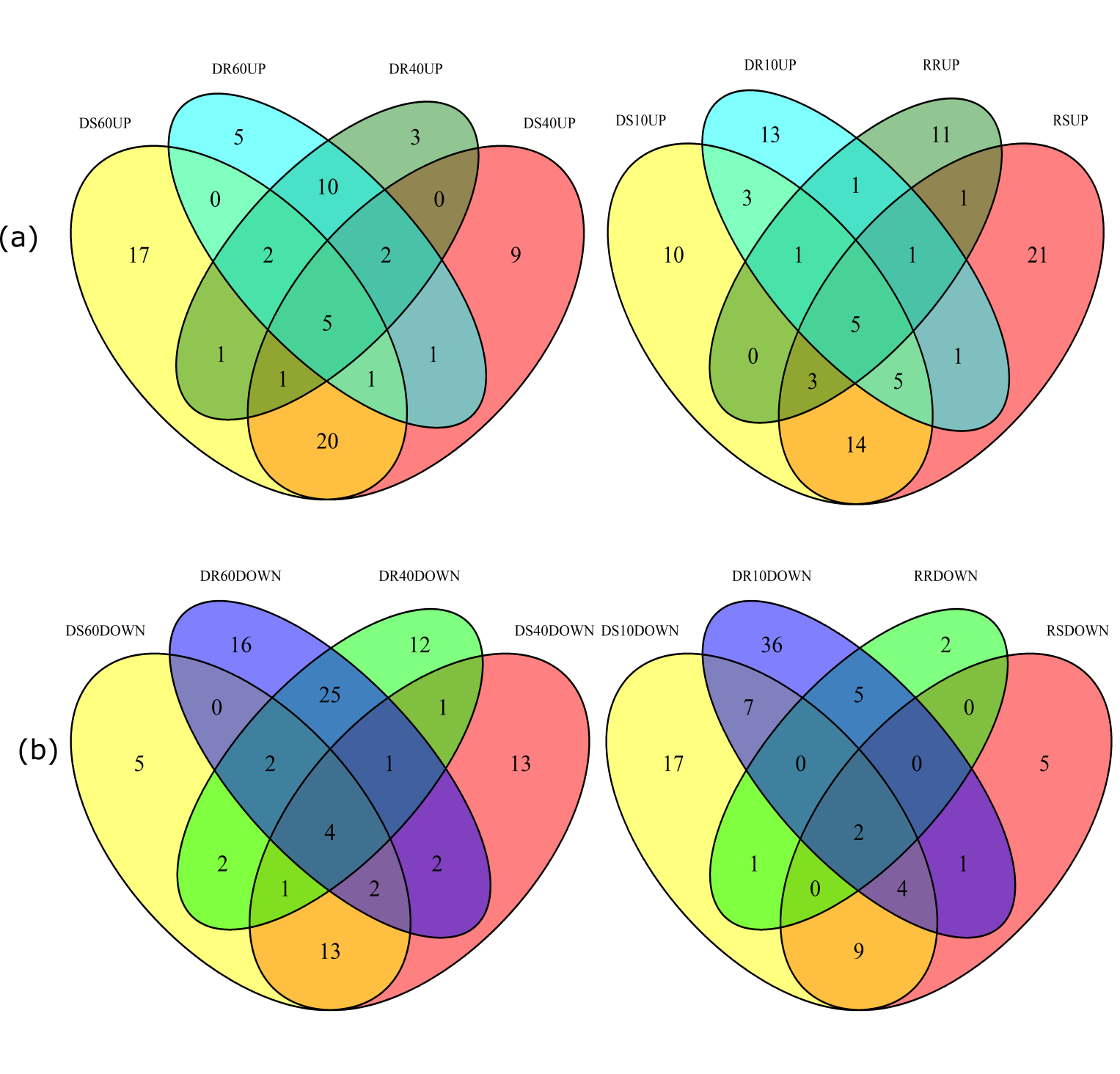

Supplement: Supplementary file 1 [file plants-07-00068-s001.zip › supplementary_figures/Figure S2.png]

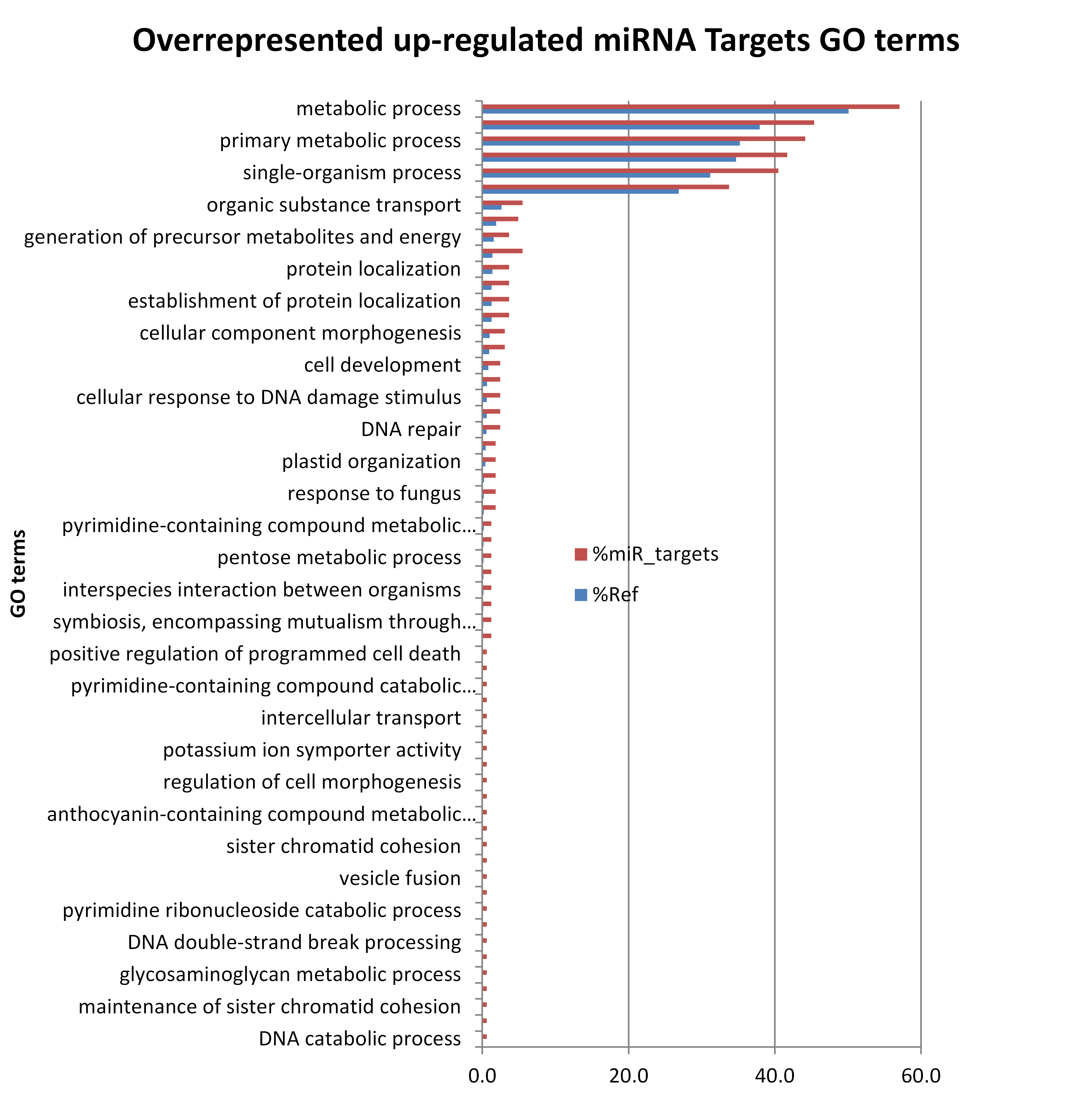

Supplement: Supplementary file 1 [file plants-07-00068-s001.zip › supplementary_figures/Figure S3.png]

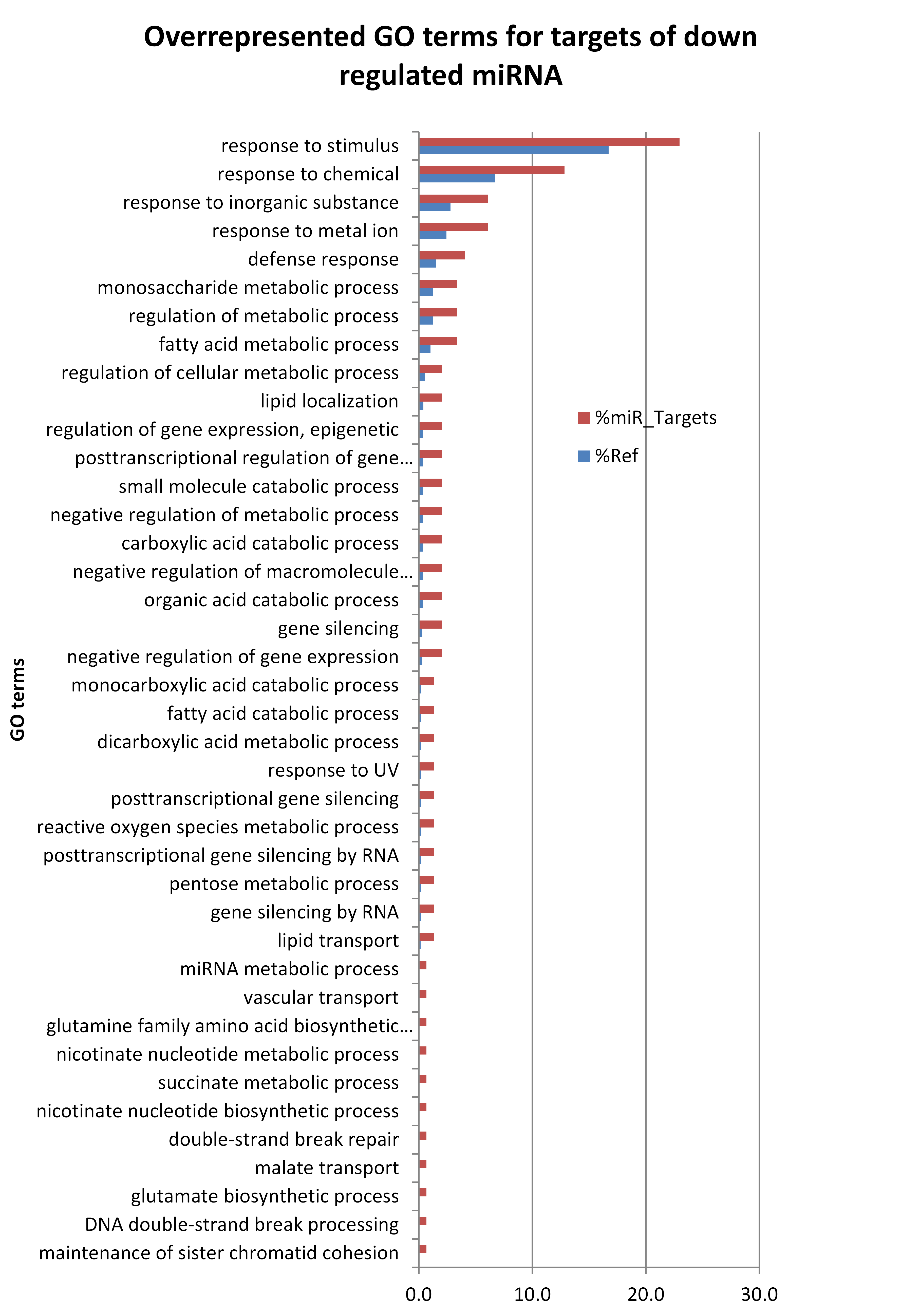

Supplement: Supplementary file 1 [file plants-07-00068-s001.zip › supplementary_figures/Figure S4.png]

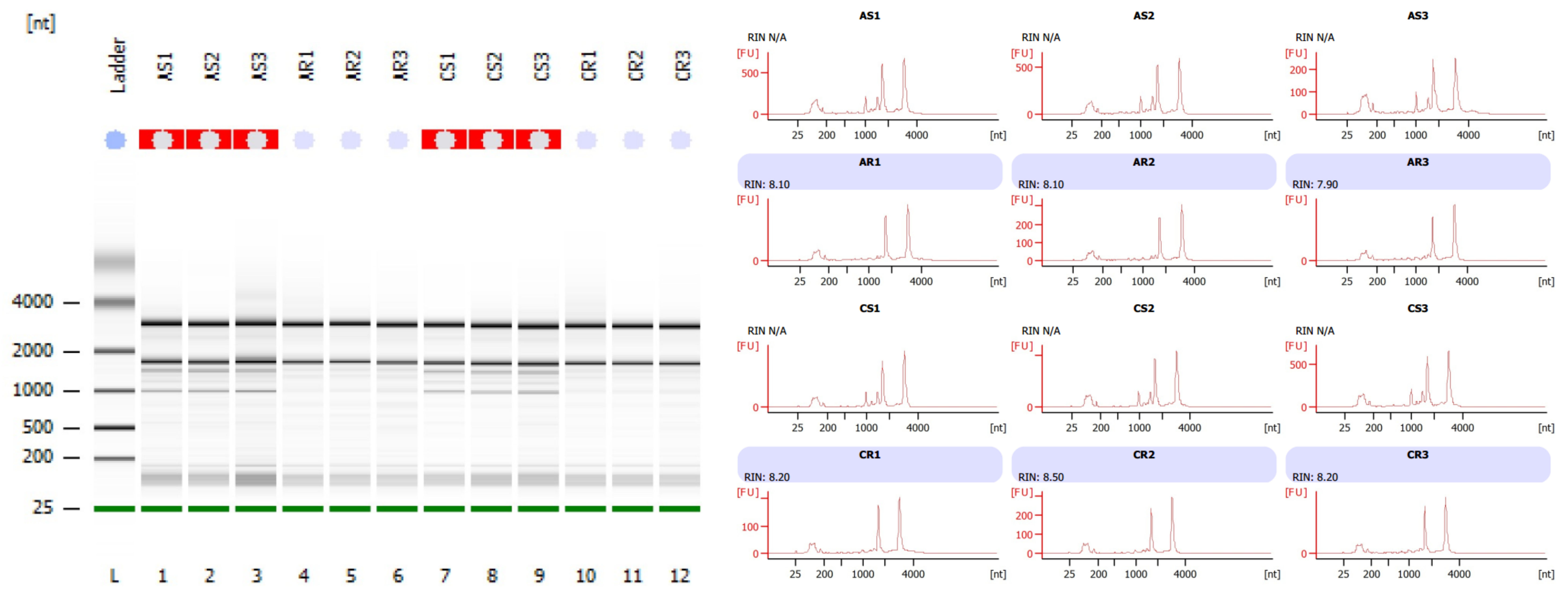

Supplement: Supplementary file 1 [file plants-07-00068-s001.zip › supplementary_figures/Figure S5.png]
